# A highly efficient, stable, durable, and recyclable filter fabricated by femtosecond laser drilling of a titanium foil for oil-water separation

**DOI:** 10.1038/srep37591

**Published:** 2016-11-21

**Authors:** Sen Ye, Qiang Cao, Qingsong Wang, Tianyuan Wang, Qing Peng

**Affiliations:** 1 Laser Micro/Nano Fabrication Laboratory, School of Mechanical Engineering, Beijing Institute of Technology, Beijing 100081, People’s Republic of China; 2 School of Power and Mechanical Engineering, Wuhan University, Wuhan 430072, China; 3 Nuclear Engineering and Radiological Sciences, University of Michigan, Ann Arbor, MI 48109, USA; 4 Department of Mechanical, Aerospace and Nuclear Engineering, Rensselaer Polytechnic Institute, Troy, NY 12180, USA

## Abstract

It has been a long standing challenge to efficiently separate oil and water since prehistoric times, and now it has become even more desirable in oily wastewater purification and oil spill cleanup. Here we introduce a super oil–water separation filter with superhydrophilicity and underwater superoleophobicity, fabricated using femtosecond laser micro-hole drilling of a titanium foil. Such a simply-made filter, without any modification, can achieve a separation efficiency exceeding 99% in eight typical oil–water mixtures. It remains highly efficient after 40 cycles of recycling and after suffering erosion by corrosive media. Furthermore, the used filter, polluted with oil, could be recovered by ultraviolet illumination. The flux of filtered water is tunable by simply selecting the aperture of the microhole or the spacing between adjacent microholes. Such advanced functionality is due to roughness and the TiO_2_ layers on the ablated surface during fabrication. With superhydrophilic and superoleophobic surfaces, this oil-water filer is also suitable for applications in anti-fouling, anti-smudge, anti-fog, and self-cleaning.

In recent years, oil–water separation has become a global challenge because of increasing amounts of industrial oily wastewater and the occurrence of oil spill accidents^1^. Inspired by fish scales and clam shells, which have excellent underwater oil–repellent properties, researchers have shown that a superhydrophilic surface usually shows superoleophobic function in aqueous solution^2^. Further studies indicated that such superhydrophilic and underwater superoleophobic surfaces have potential uses in oil–water mixtures separation and oil-and-water emulsions separation^3^,^4^,^5^,^6^,^7^,^8^,^9^,^10^. Key points in producing a superhydrophilic surface are constructing micro/nanoscale rough structures on substrates and improving the free energy of surface^11^,^12^,^13^,^14^,^15^. For example, it is reported to coat a hydrophilic organic polymer with high free energy on steel mesh to produce a membrane with a rough micro/nano structure^3^,^16^,^17^. Such membranes can separate water from different oil–water mixtures with efficiencies exceeding 99%. However, most of the polymer-coated membranes could not be used in industrial wastewater treatment because the organic coatings would become unstable when exposed to harsh conditions^18^. Another material, TiO_2,_ is a stable and hydrophilic coating material^18^,^19^, and it has been shown that the as-coated TiO_2_ could catalyze the degradation of organic pollutants with ultraviolet (UV) illumination. However, almost all of the methods used for producing such TiO_2_-coated membranes are chemical approaches^18^,^19^,^20^,^21^,^22^, which usually need two or more steps that are time-consuming, and the chemical reagents are not environmentally friendly. In addition to chemical methods, oil-water separation filters were fabricated using a nanosecond laser to drill holes on a copper foil^23^. However, the heat effects of nanosecond laser may induce deformations, thus reducing the reliability.

The femtosecond laser, a promising tool, has some excellent characteristics in the ablation of micro/nanoscale hierarchical rough structures^24^,^25^,^26^,^27^,^28^, such as non-contact manufacturing, a small heat-affected zone, high precision and non-polluting processes^29^,^30^,^31^. Chen *et al*. used a femtosecond laser to fabricate a rough micro-mountain array structure and a layer of TiO_2_ on a substrate of Ti^32^. After UV illumination of this structure, the properties of the irradiated area changed from superhydrophobic (underwater superoleophilic) to superhydrophilic (underwater superoleophobic) due to the photo-induced hydrophilicity of the TiO_2_ layer. However, this functional surface cannot let the aqueous solution pass, for the simple reason that it has no through-hole, thus the micro-mountain array can only function as anti-fouling surface, but not as filter for oil-water separation. Inspired by their work, we developed a novel method for high-efficiency and high-stability oil–water separation, which entails fabricating a microhole array instead of micro-mountain array on a titanium foil using a femtosecond laser. The fabricated through-hole array on the titanium foil can not only inherit the properties of micro-mountain array, but also offer pathways for the aqueous solution passing through. Furthermore, by controlling the surface morphology during femtosecond laser ablation, our oil-water separation filter can be highly efficient, stable and recyclable.

Our method is a simple and elegant one-step strategy. Controlling the aperture and the spacing of the ablated microhole array, we obtained an optimized sample, which showed outstanding underwater superoleophobic properties and could effectively separate water from various oil–water mixtures.

## Results

### Morphology and chemical composition of prepared filters

**Figure 1:**
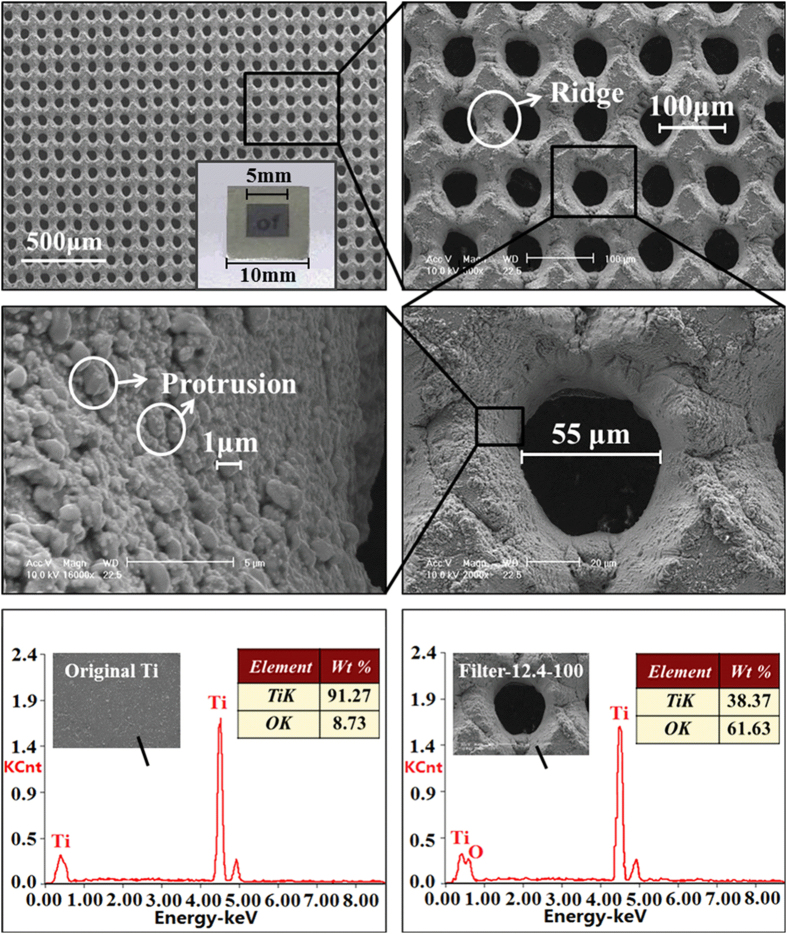
Surface morphology and chemical composition of filter-12.4-100. (**a–d**) SEM images of ablated titanium foil fabricated with a laser fluence of 12.4 J/cm^2^ and a microhole spacing of 100 μm (filter-12.4-100). The inset in (**a**) is the photograph of filter-12.4-100. (**a**) Large-area view of filter-12.4-100. (**b**) Further magnified image of filter-12.4-100. (**c**) Enlarged view of a single microhole on filter-12.4-100. (**d**) Higher magnification image of the microhole wall. (**e**) EDXS result of the original Ti surface. (**f**) EDXS result of the ablated area on filter-12.4-100.

Figure 1a–d show optical photograph and SEM images of the ablated titanium foil fabricated with a laser fluence of 12.4 J/cm^2^ and a microhole spacing of 100 μm (filter-12.4-100). The filter has the effective area of 5 × 5 mm^2^ located in the center of a titanium foil (10 × 10 × 0.1 mm^3^), and it exhibits good light transmittance due to the through-hole array structure (inset in Fig. 1a). There is an array of micro funnel-shaped through hole on the filter, with the aperture of microholes about 55 μm. Such funnel shape was due to Gaussian laser ablation^33^,^34^. The edges of adjacent microholes overlapped partially and formed a ridge structure. A further magnified image showed that the microhole wall was covered by irregular protrusions ranging in size from hundreds of nanometers to several microns (Fig. 1d). These microscale or nanoscale protrusions were shaped by the resodification of ejected materials during the laser ablation process^35^, and significantly increase the surface area of hole sidewall, which played an important role in the oil-water separation. Figure 1e and f show EDXS results for the titanium foil before and after femtosecond laser ablation. The original foil is composed primarily of Ti. Upon femtosecond laser ablation, the atomic proportion of Ti decreased from 91.27% to 38.37%, whereas the atomic proportion of oxygen increased from 8.73% to 61.63%. These results indicate that the laser-ablated Ti surface was oxidized during the formation of the microhole array, resulting in a rough TiO_2_ layer in the ablated area.

The density of microholes, reflecting the number of holes in a unit area (1 mm^2^), can be controlled precisely by microhole spacing. When the spacing was adjusted from 100 to 300 μm, the density of microholes decreased from 100 mm^−2^ to 9 mm^−2^, and the non-ablated area between microholes increased significantly (see Supplementary Fig. S1). When the spacing was set lower than 100 μm, for example 50 μm, the microholes would connect with one another. It is worth noting that this metal foil with microholes is highly mechanically durable for use in oil-water separation.

The aperture of the microhole can be modulated systematically by simply changing the laser fluence. As shown in Fig. S2 in the Supplementary Information, at the same microhole spacing, the aperture of the microholes increased almost linearly with the fluence from 3.1 to 15.5 J/cm^2^. The increased aperture inhibited the growth of ridge structure in the overlapped area, and indirectly caused that more ejected materials managed to experience resolidification, which further improved the micro/nanoscale structure on the ablated surface. Moreover, changes in the spacing and fluence did not affect the content of titanium and oxygen in the ablated region (see Supplementary Fig. S3), indicating that there was always a layer of TiO_2_ in the ablated area.

### Wetting properties of prepared filters

**Figure 2:**
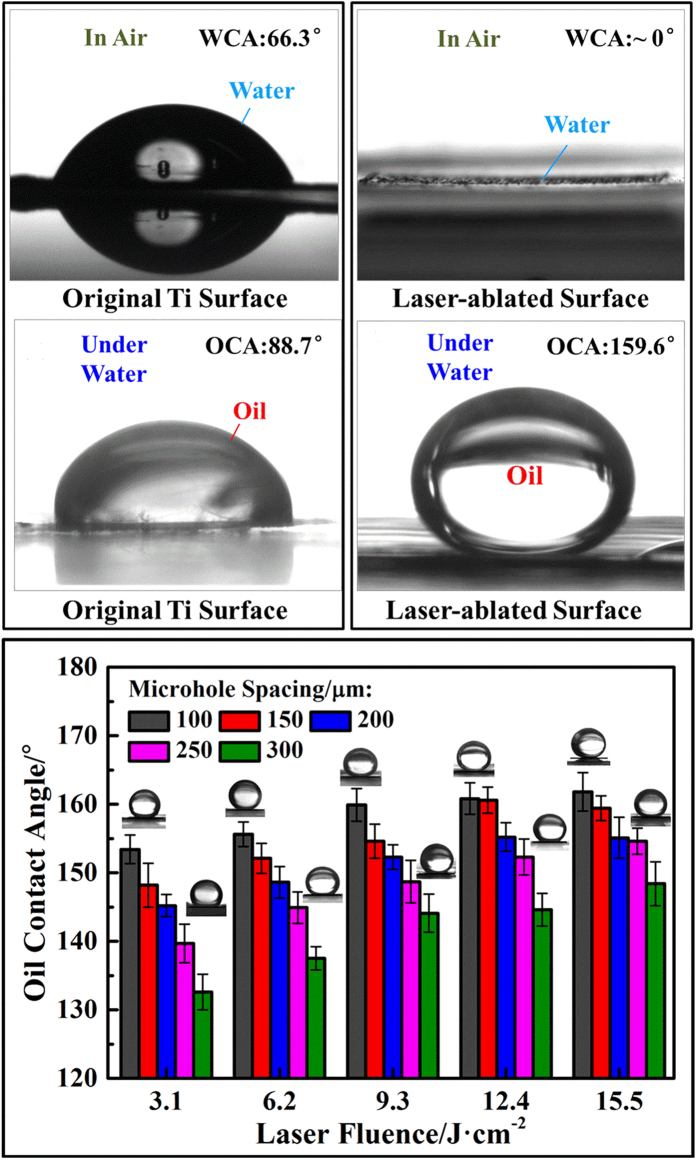
Wetting properties of original titanium foil and prepared filters. (**a**) A water droplet (5 μL) in air and (**c**) an oil droplet (1, 2-dichloroethane, 5 μL) in water on the original Ti surface with WCA of 66.3 ± 2.1° and OCA of 88.7 ± 1.4°. (**b**) A water droplet in air and (**d**) an oil droplet in water on filter-12.4-100 with WCA near 0° and OCA of 159.6 ± 2.1°. (**e**) Statistical graph of underwater OCA on different ablated surfaces. The above five insets are photographs of oil droplets on the ablated surface with a microhole spacing of 100 μm and laser fluences from 3.1 to 15.5 J/cm^2^. The lower five insets are photographs of oil droplets on the ablated surface with a microhole spacing of 300 μm and laser fluences from 3.1 to 15.5 J/cm^2^.

The wettability of solid surfaces depends strongly on both the geometrical structure and the chemical composition^11^. Thus, the micro/nanoscale rough structures and the TiO_2_ layer on the ablated surface significantly influence the wetting properties of the prepared filter. Here, we took filter-12.4-100 as a representative and compared its wettability with that of the original titanium foil. As shown in Fig. 2, in an air environment, the original titanium foil, with a surface water contact angle (WCA) of 66.3 ± 2.1°, exhibited weak hydrophilic properties. However, when the water droplet contacted filter-12.4-100, it quickly spread and penetrated, resulting in a WCA near 0°. This indicated that filter-12.4-100 showed superhydrophilic properties. In a water environment, the original titanium foil, with a surface oil contact angle (OCA) of 88.7 ± 1.4°, showed weak underwater oleophilic properties, whereas filter-12.4-100, with a surface OCA as high as 159.6 ± 2.2°, exhibited underwater superoleophobic properties. Moreover, when the filter was slightly tilted, the oil droplet would roll off the surface (see Supplementary Fig. S4 and Movie S1). According to Yong’s research^36^, our prepared filter has ultralow oil-adhesion.

According to Jiang’s model^2^, the underwater contact angle of an oil droplet on a solid surface can be described by Equation (1):


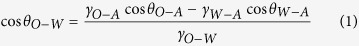


where *γ*_*O−A*_, *γ*_*W−A*_, and *γ*_*O−W*_ are the interface tensions of oil/air, water/air, and oil/water, respectively, *θ*_*O−A*_ and *θ*_*W−A*_ are the contact angles of oil and water droplets in air, respectively, and *θ*_*O−W*_ is the contact angle of an oil droplet in water. It is known that *γ*_*O−A*_ is generally 20–30 mN∙m^−1^, and *γ*_*W−A*_ is 73 mN∙m^−1 ^15,37. Because *θ*_*O−A*_ is very small, usually near 0°, a decrease in *θ*_*W−A*_ leads to a larger *θ*_*O−W*_. That is, a more hydrophilic substrate becomes more oleophobic underwater. After femtosecond laser ablation, micro/nanoscale rough structures and a hydrophilic TiO_2_ layer were formed on the Ti substrate (Fig. 1). The rough TiO_2_ layer increased the free energy of the ablated surface. Besides, the funnel shape of the hole sidewall provided large hydrophilic surface areae^18^,^19^. Both further improved the hydrophilic properties of filter-12.4-100 and resulted in the significant decrease in *θ*_*W−A*_. Thus, when the filter was immersed in water, a high volume of water would be trapped by the micro/nanoscale rough structures, reducing the contact area between the oil and the surface. Under this condition, the *θ*_*O−W*_ on the ablated surface would increase significantly. Thus, filter-12.4-100 showed excellent underwater superoleophobic properties.

Further measurements show that, with greater laser fluence and reduced microhole spacing, the water droplet spread faster (see details in Supplementary Movie S2), and the underwater oil contact angle was larger (Fig. 2e). Generally, higher laser fluence and smaller microhole spacing produced larger micro/nanoscale rough structure and oxidized region on the ablated surface. This rough structure would further amplify the hydrophilic properties of the TiO_2_, resulting a filter with better hydrophilicity and underwater oleophobicity.

### Oil-water separation with filter-12.4-100

**Figure 3:**
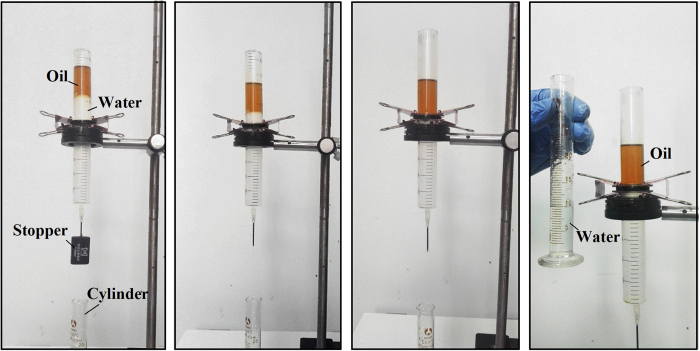
Oil–water separation study with filter-12.4-100. (**a**) A mixture of sesame oil and water was poured into the upper plastic tube. (**b**) Gravity induced water to permeate through the filter and flow into the cylinder. (**c**) Oil was intercepted and kept in the upper tube. (**d**) Pure water was successfully separated from the mixture, and no oil was observed in the collected water.

Based on above results and discussion, filter-12.4-100, which exhibited remarkable superhydrophilic and underwater superoleophobic properties, is suitable for oil–water separation. An experimental oil–water separation was carried out using the set-up illustrated in Fig. 3. A pre-wetted filter-12.4-100 was sandwiched between two plastic tubes. First, a mixture of sesame oil and water (50%, v/v) was poured into the upper tube (Fig. 3a). Then, the stopper was opened. Water, with a higher density than the oil, quickly permeated through the filter and dropped into the cylinder below (Fig. 3b). The oil was retained above the filter because of its underwater superoleophobic properties (Fig. 3c). Finally, pure water was separated from the oil–water mixture by gravity alone. No oil could be seen in the collected water (Fig. 3d). A video illustrating the separation of sesame oil and water is provided as Supplementary Movie S3.

## Discussion

**Figure 4:**
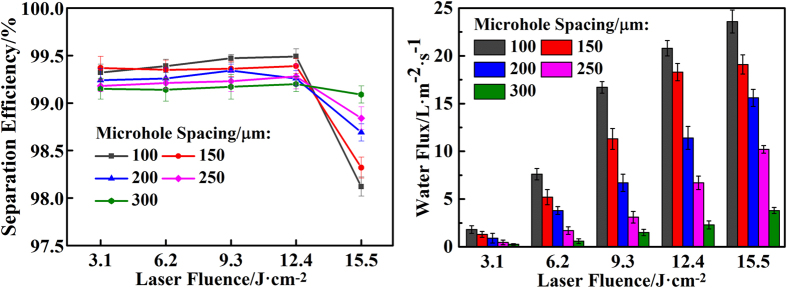
Oil–water separation efficiency and velocity of prepared filters. (**a**) Separation efficiency of different filters in terms of the oil rejection coefficient. (**b**) Separation velocity of different filters in terms of the water flux.

Several more analyses and measurements were made to demonstrate the high efficiency of the method. First, the separation efficiency can be calculated with the equation η = (m_1_/m_0_) × 100^38^, where m_0_ and m_1_ are the masses of oil before and after the separation process, respectively. As shown in Fig. 4a, when the laser fluence was 3.1–12.4 J/cm^2^ (corresponding aperture was 15–55 μm), for the prepared filters with different microhole spacings (100–300 μm), the separation efficiency always exceeded 99%. However, when the laser fluence increased to 15.5 J/cm^2^ (corresponding aperture was 70 μm), the separation efficiency of prepared filters decreased notably, particularly when the microhole spacing is 100 μm. This is because the aperture of the filter is relatively too large to suffer enough intrusive pressure (see Supplementary Fig. S5). As a consequence, water and oil passed simultaneously through the filter, resulting in a significant decline in separation efficiency. The above results demonstrate that filter-12.4-100, with efficiency up to 99.5%, is good for oil–water separation.

Second, the separation velocity can be measured by the flux of the filtered water. The flux is calculated with the equation F = V/St, where V is the volume of water that permeates through the filter. We fixed V at 10 mL. S is the area of the filter, and t is the required time for complete separation. As shown in Fig. 4b, with higher laser fluence (3.1–15.5 J/cm^2^) and a smaller microhole spacing (100–300 μm), the flux of filtered water was larger, indicating that the as-prepared filter exhibited higher separation velocity. The reason for this is that a higher laser fluence and a smaller microhole spacing produced larger aperture and higher density of microholes, which is more beneficial to the flow of the liquid. Further experiment showed that the large-aperture filter could be used for a continuous and long-term oil-water separation (see Supplementary Information Movie S4). These results demonstrate that filter-12.4-100, with water flux up to 20 L∙m^−2^∙s^−1^, is capable of separating a large amount of oil-water mixtures.

**Figure 5:**
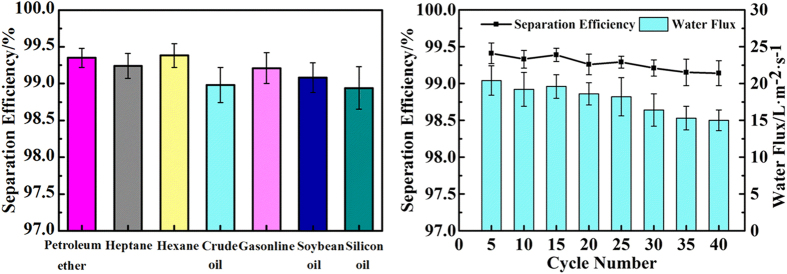
Appling and recycling tests with filter-12.4-100. (**a**) Separation efficiency of filter-12.4-100 for various oil–water mixtures. (**b**) Filter-12.4-100 retained high separation efficiency and water flux after using it 40 times.

Third, the applicability of filter-12.4-100 was assessed. As shown in Fig. 5a, various organic solvent-water mixtures and oil–water mixtures, including petroleum ether, heptane, hexane, crude oil, gasoline, soybean oil and silicon oil, were successfully separated with efficiencies all exceeding 99%.

Fourth, the anti-corrosion performance of filter-12.4-100 was studied. The filter was immersed in corrosive media of 1 M HCl, 1 M KOH, and 1 M NaCl for 24 h, respectively. Figure S6a† and S6b† show the SEM images and EDXS pattern of filter-12.4-100 after the corrosion tests. Neither the micro/nanoscale structure nor the chemical composition of the filter surface was destroyed by the corrosive solutions. Contact angles of water and oil were also measured (see Supplementary Fig. S6c and S6d). The WCA in air was near 0°, and the underwater OCA was above 150°, indicating that the filter still retained high hydrophilicity and underwater oleophobicity. After being immersed in corrosive media, the filter still could separate water from the oil–water mixture with efficiency exceeding 99% (see Supplementary Fig. S6e–g).

Finally, the self-cleaning properties and recyclability of filter-12.4-100 were assessed. When the filter was contaminated by oleic acid, the contaminated surface exhibited hydrophobic and underwater oleophilic properties with a WCA of 119.4 ± 1.8° and an underwater OCA of 21.6 ± 2.1° (see Supplementary Fig. S7a). Such wetting characteristics resulted in the contaminated filter losing its oil–water separation ability (see Supplementary Fig. S7b–d). After irradiation with UV light for 1 h, the WCA on the irradiated surface decreased to 0°, and the underwater OCA increased to 160° (see Supplementary Fig. S7e). Moreover, the irradiated filter reacquired its oil–water separation abilities (see Supplementary Fig. S7f–h). According to current research, under UV illumination, the laser-induced TiO_2_ layer on the filter could generate photo-electrons and holes, which then reacted with oxygen and water to produce highly reactive species, such as superoxide anions and hydroxyl radicals^39^,^40^. These highly reactive species could then decompose and remove the organic contaminants, leading to restoration of the hydrophilicity and underwater oleophobicity of the irradiated surface. Thus, even if filter-12.4-100 suffered contamination by organisms, the contaminated filter could be recovered after UV illumination. Such self-cleaning properties demonstrated that filter-12.4-100 possessed excellent recyclability. Even after recycling 40 times, filter-12.4-100 still could separate water from oil–water mixture with efficiency as high as 99% and water flux exceeding 10 L∙m^−2^∙s^−1^ (Fig. 5b). The decrease of separation efficiency and water flux may result from a small amount of residual pollutants which exist on the non-ablated area between adjacent microholes.

These results demonstrated that filter-12.4-100 possesses excellent applicability, stability, durability, and recyclability, making it suitable for applications in, for example, oil pollution clean-up, sewage treatment, anti-fouling, anti-smudge, anti-fog, and self-cleaning^15^.

In summary, we developed a one-step method to produce high-efficiency and high-stability oil–water separation filters based on femtosecond laser micro-hole drilling of titanium foils. Such filters exhibited excellent superhydrophilic and underwater superoleophobic properties due to the micro/nanoscale rough structures as well as the TiO_2_ layer on the surfaces. The separation efficiency and velocity depend on the aperture and density of microholes. These parameters can be controlled by the laser fluence and microhole spacing. The filter fabricated at a laser fluence of 12.4 J/cm^2^ and a microhole spacing of 100 μm (filter-12.4-100) achieved separation efficiencies up to 99.5% and a water flux up to 20 L∙m^−2^∙s^−1^. An anti-corrosion experiment showed that the filters exhibited excellent stability. Moreover, the laser-induced TiO_2_ layer on the ablated surfaces could catalyze the degradation of intrusive organic pollutants with UV illumination. Such self-cleaning properties ensured that the filters could be recycled at least 40 times. Thus, our method is suitable for applications in oil pollution cleanup, sewage treatment, anti-fouling, anti-smudge, anti-fog, and self-cleaning.

## Methods

### Preparation of oil–water separation filter

**Figure 6:**
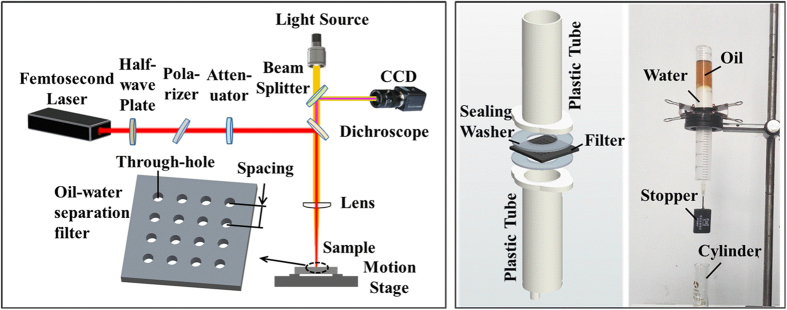
Experimental setups for preparation filters and for oil-water separation. (**a**) Experimental optical path of femtosecond laser micro-hole drilling of the titanium foil; the inset shows a schematic of the prepared filter. (**b**) Diagrammatic sketch (left) and real-object picture (right) of the home-made device used for oil–water separation.

A schematic of the femtosecond laser ablation is shown in Fig. 6a. A titanium foil (10 × 10 × 0.1 mm^3^) was mounted on a motion stage and then irradiated by an amplified commercial Ti:sapphire laser system (Spectra-Physics). Upon femtosecond laser irradiation, a micro through-hole array was formed in the foil (Fig. 1a). The femtosecond laser has a central wavelength of 800 nm, a pulse width of 50 fs (FWHM), and a repetition rate of 1 kHz. The focused spot size of the laser beam with a plano-convex lens was 64 μm. The laser energy fluence could be varied with the attenuator from 3.1 to 15.5 J/cm^2^; thus, the aperture of the microhole could be controlled from 15 to 70 μm. The spacing of the microhole array could be adjusted from 100 to 300 μm using the motion stage. After irradiation, the samples were cleaned ultrasonically with acetone, ethanol, and deionized water for 5 min, respectively.

### Characterization

The ablated surface was observed using a Hitachi S4800 scanning electron microscope (SEM). The chemical composition was analyzed by energy dispersive X-ray spectroscopy (EDXS). The static and dynamic wettability of the samples were investigated using a video-based optical contact angle-measuring device (OCA 15 Plus; Data Physics Instruments) and a sessile drop technique. An organic analytical reagent, 1,2-dichloroethane, was used as the test oil after it was dyed with Sudan III. The volume of the oil and water droplet was set at 5 μL. Average values were obtained by measuring five different points on the same surface.

### Oil–water separation

The oil–water separation was carried out with the home-made device illustrated in Fig. 6b. The sample, sealed by two washers, was sandwiched between two plastic tubes with a diameter of 20 mm. The oil–water mixture (50%, v/v) was poured into the upper tube. A piece of rubber, used as a stopper, was fixed on a needle connected to the lower tube. A cylinder placed at the bottom of the whole device was used to collect the separated aqueous solution. When the stopper was opened, the water solution would quickly penetrate the sample and drop into the cylinder, whereas the oil layer would remain above the sample, resulting in the complete separation of oil and water.

## Additional Information

**How to cite this article**: Ye, S. *et al*. A highly efficient, stable, durable, and recyclable filter fabricated by femtosecond laser drilling of a titanium foil for oil-water separation. *Sci. Rep*. **6**, 37591; doi: 10.1038/srep37591 (2016).

**Publisher’s note**: Springer Nature remains neutral with regard to jurisdictional claims in published maps and institutional affiliations.

## Supplementary Material

Supplementary Information

Supplementary Movie S1

Supplementary Movie S2

Supplementary Movie S3

Supplementary Movie S4
