# Supplementary material for: A highly efficient, stable, durable, and recyclable filter fabricated by femtosecond laser drilling of a titanium foil for oil-water separation
Source: Sci Rep. 2016 Nov 21;6:37591. doi: 10.1038/srep37591 (PMC5116619; doi:10.1038/srep37591)
Supplement: Supplementary Information [file srep37591-s1.pdf]

Electronic Supplementary Information

A highly efficient, stable, durable, and recyclable filter fabricated by femtosecond laser drilling of a titanium foil for oil-water separation

Sen Ye,[a] Qiang Cao,*[a] Qingsong Wang,[a] Tianyuan Wang,[a] and Qing Peng*[b,c,d]

[a] *Laser Micro/Nano Fabrication Laboratory, School of Mechanical Engineering, Beijing Institute of Technology, Beijing 100081, People's Republic of China*
[b] *School of Power and Mechanical Engineering, Wuhan University, Wuhan 430072, China.*
[c] *Nuclear Engineering and Radiological Sciences, University of Michigan, Ann Arbor, MI 48109, USA.*
[d] *Department of Mechanical, Aerospace and Nuclear Engineering, Rensselaer Polytechnic Institute, Troy, NY 12180, USA*
E-mail: caoqiang@bit.edu.cn, qpeng.org@gmail.com

This file includes Experimental details, Supplementary Fig. S1-S7, Legend for Supplementary Information Multimedia Movie S1, Movie S2, Movie S3 and Movie S4.

Preparation of underwater superoleophobic filters with different microhole

apertures and microhole spacings: The underwater superoleophobic filters were fabricated by femtosecond laser micro-hole drilling of titanium foil in air environment. The micro through-hole array was prepared by a point to point ablation. The laser fluence could be varied with the attenuator from 3.1 to 15.5 J/cm^2; thus, the aperture of the microhole could be controlled from 15 to 70 μm. The spacing of the microhole array could be adjusted from 100 to 300 μm using the motion stage. The processed area was held constant at 5×5 mm^2. After ablation, the samples were cleaned ultrasonically with acetone, ethanol and deionized water for 5 minutes, respectively.

Corrosion resistance test: The anti-corrosion performance of filter fabricated with laser fluence of 12.4 J/cm^2 and microhole spacing of 100 μm (filter-12.4-100) was studied by immersing the filter into corrosive medium of 1 M HCl, 1 M KOH, 1 M NaCl for 24h, respectively. The morphologies and chemical composition of the filter were analyzed using scanning electron microscope (SEM) and energy dispersive X-ray spectroscopy (EDXS), respectively. After each immersion with different corrosive solutions, water contact angle (WCA) in air and oil contact angle (OCA) in water were measured to evaluate the wetting properties of the filter. Moreover, after being immersed into corrosive media, the filter was used for oil-water separation to compare its separation ability with the fresh-prepared filter-12.4-100.

Self-cleaning experiment: Filter-12.4-100 was contaminated with oleic acid by immersing the filter into a 1.0% (v/v) acetone solution of oleic acid for 20 min. Then the filter was subject to UV illumination for 1 h. Water contact angles in air and oil contact angles in water before and after UV irradiation were measured to demonstrate the variation of wetting properties of filter. Moreover, the contaminated filter as well as the UV-irradiated filter was respectively used to separate water from sesame oil-water mixture to compare their separation ability.

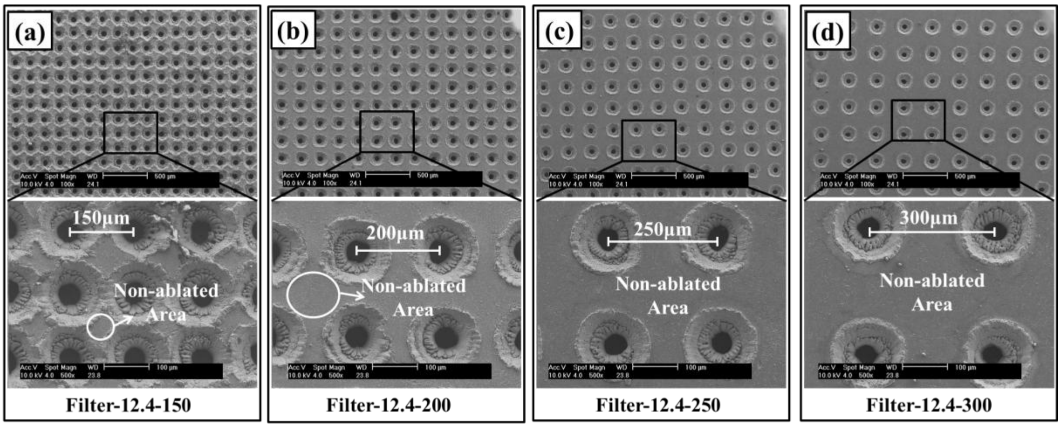

Fig. S1 SEM images of ablated titanium foils fabricated with laser fluence of 12.4 J/cm^2 and microhole spacing of (a) 150 μm (filter-12.4-150). (b) 200 μm (filter-12.4-200). (c) 250 μm (filter-12.4-250). (d) 300 μm (filter-12.4-300).

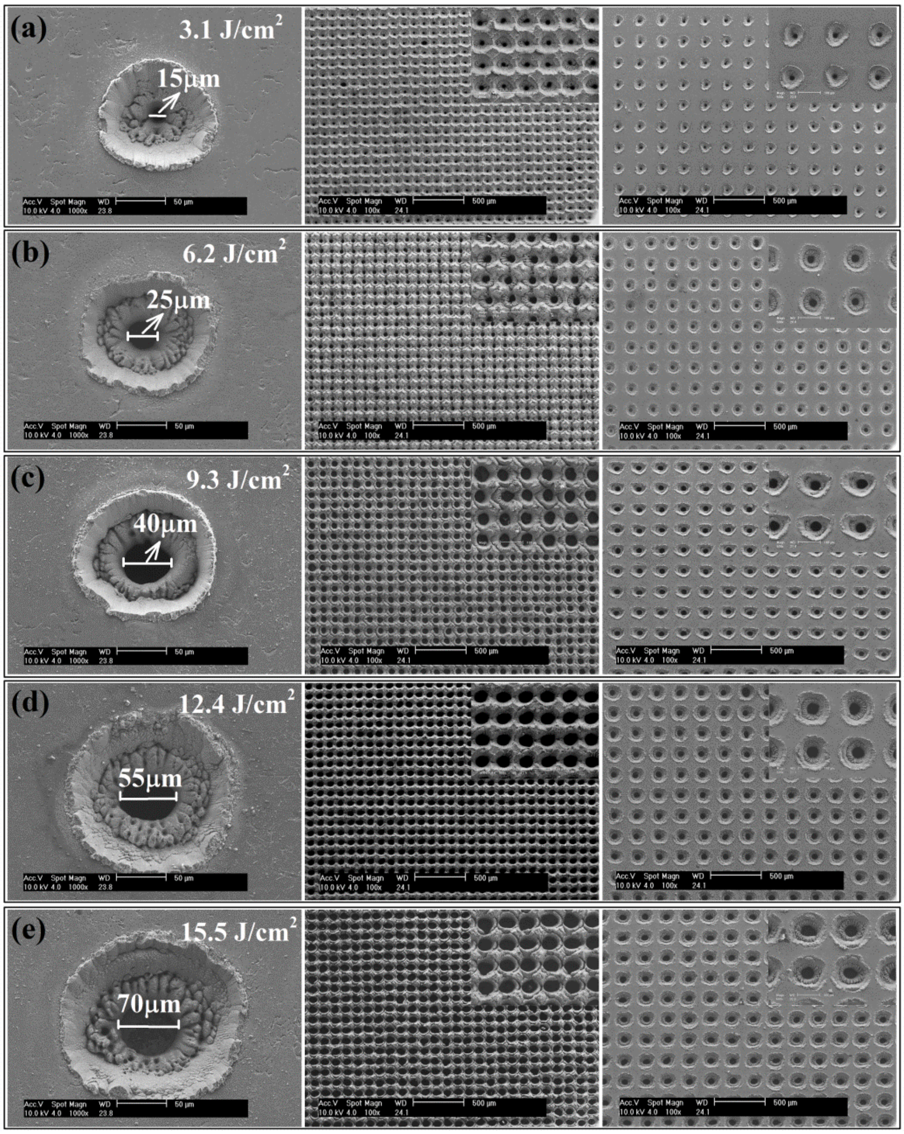

Fig. S2 SEM images of single microhole (left column), microhole array with spacing of 100 μm (middle column), microhole array with spacing of 200 μm (right column) fabricated by the laser fluence of (a) 3.1 J/cm^2, (b) 6.2 J/cm^2, (c) 9.3 J/cm^2, (d) 12.4 J/cm^2, (e) 15.5 J/cm^2. The inset of each picture in middle and right column is the magnified image of the filter.

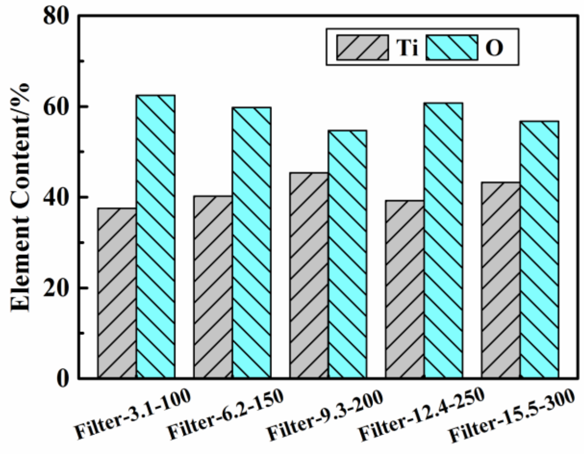

Fig. S3 Content of titanium and oxygen in ablated region on different filters. The content of titanium and oxygen has no obvious change in different ablated regions, which indicates that there was always a layer of TiO_2 in the ablated area.

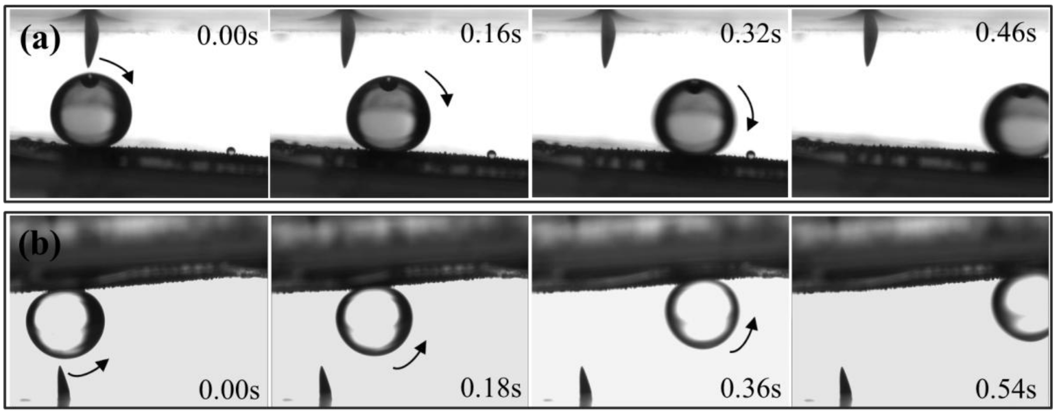

Fig. S4 (a) Heavy oil (1, 2-dichloroethane) droplet and (b) light oil (sesame oil) droplet rolling on a tilted filter-12.4-100. The sliding angle for heavy oil droplet is about 5.1° and for light oil droplet is about 4.7°. It shows that the as-prepared filter has ultralow oil-adhesion.

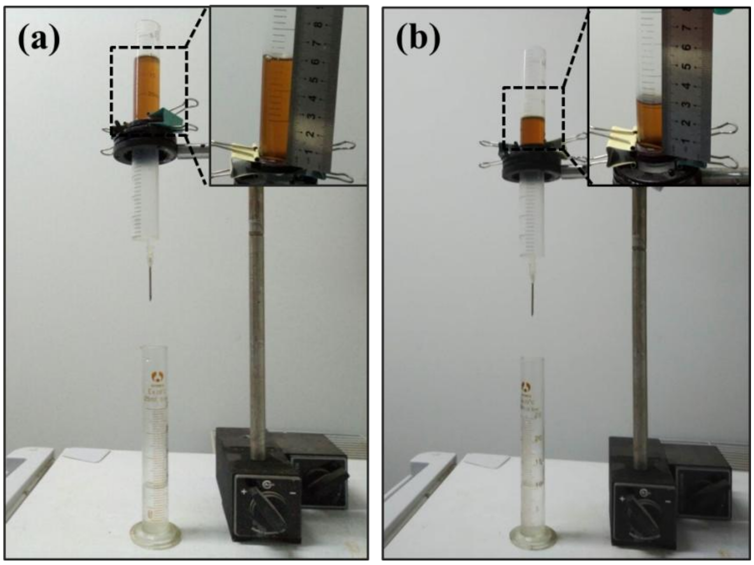

Fig. S5 Intrusive pressure test with filter-12.4-100 and filter-15.5-100. (a) The maximum height of oil filter-12.4-100 can support is about 6.1 cm, and the intrusive pressure is about 550 Pa. (b) The maximum height of oil filter-15.5-100 can support is about 2.9 cm, and the intrusive pressure is about 261 Pa. During the oil-water separation test, the height of the oil column was about 4 cm and the intrusive pressure was about 361 Pa. Filter-15.5-100 could not support such high pressure and a few oil would permeate through the filter, which further resulted in the decreasing of separation efficiency.

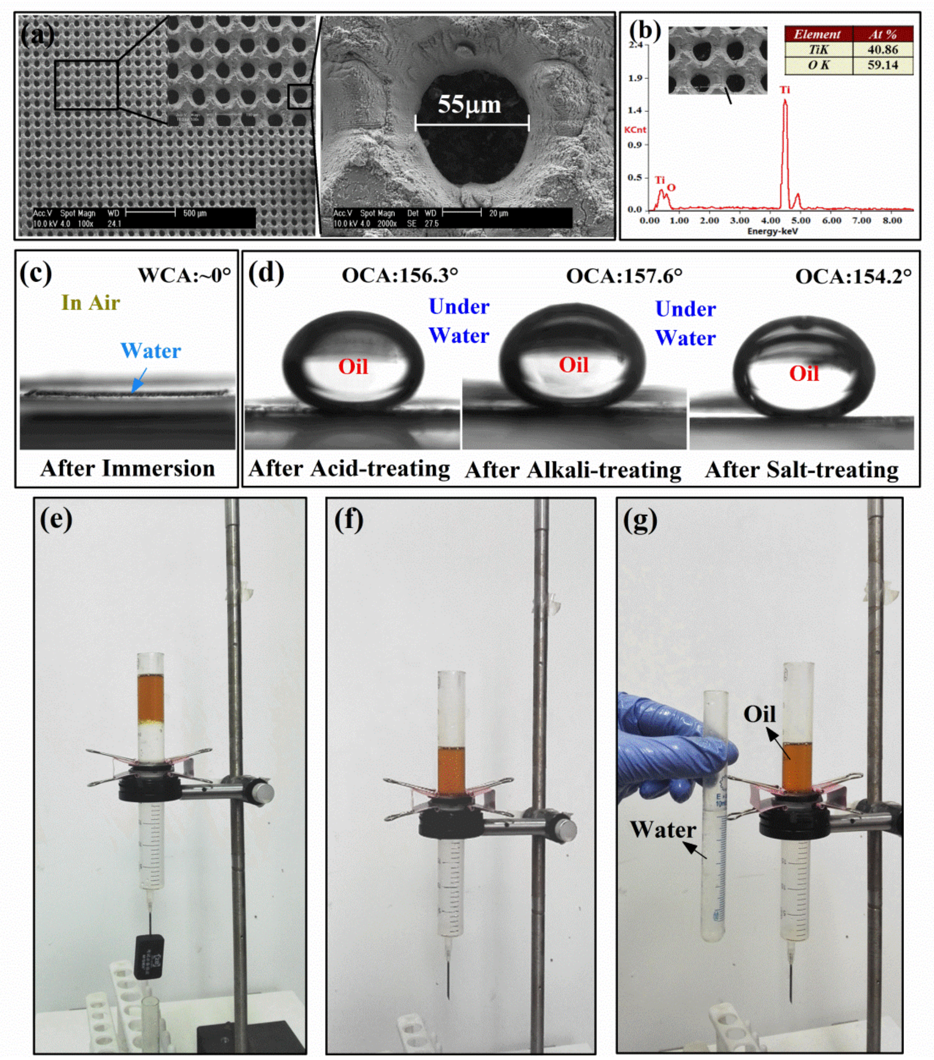

Fig. S6 (a) SEM images and (b) EDXS pattern of filter-12.4-100 after the corrosion tests. (c) Image of water droplet on the corrosive medium treated filter-12.4-100. (d) Images of oil droplets on the acid-treated filter-12.4-100, the alkali-treated filter-12.4-100 and the salt-treated filter-12.4-100, respectively. (e-g) Separation of oil-water mixture by the corrosive medium treated filter-12.4-100.

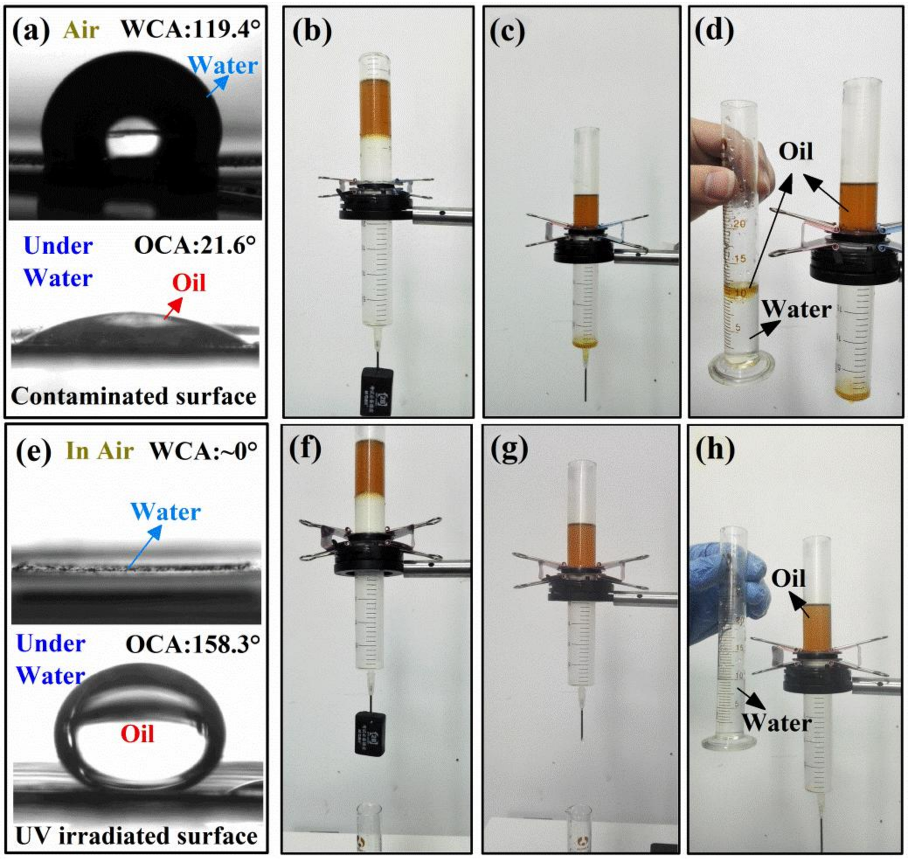

Fig. S7 (a) Photographs of water droplet (upper) and oil droplet (lower) on the oleic-acid-contaminated filter-12.4-100. The WCA and underwater OCA on the contaminated surface are 119.4° and 21.6°, respectively. (b-d) Separation of oil-water mixture by the contaminated filter-12.4-100. (e) Photographs of water droplet (upper) and oil droplet (lower) on the oleic-acid-contaminated filter-12.4-100 after the subsequent UV illumination. The WCA and underwater OCA on the UV-irradiated surface are 0° and 158.3°, respectively. (f-h) Separation of oil-water mixture by the UV-irradiated filter-12.4-100.

Supplementary movie captions:

Movie S1: Rolling behavior of oil droplet on filter-12.4-100 in water environment. When the filter was slightly tilted, the oil droplet would roll off the surface, no matter for heavy oil droplet or light oil droplet. It indicated that the as-prepared filter has ultralow oil-adhesion.

Movie S2: Permeating behavior of water droplet on different filters (filter-15.5-100, filter-9.3-200 and filter-3.1-300). In summary, with a larger fluence and a smaller microhole spacing, the as-prepared filter exhibited better hydrophilic property and water droplet on the surface spread faster.

Movie S3: Oil-water separation by filter-12.4-100. The pre-wetted filter was fixed between two plastic tubes (diameter ~ 20 mm). A mixture of sesame oil and water (50%, v/v) was poured into the upper plastic tube. When the stopper was opened, water quickly passed through the filter and dropped into the cylinder, whereas the sesame oil was retained in the upper plastic tube. Finally the water was successfully separated from the oil-water mixture, and no oil could be seen in the collected water.

Movie S4: A continuous and long-term oil-water separation by filter-12.4-100. The pre-wetted filter was fixed between two plastic tubes (diameter ~ 30 mm). Large amount of oil-water mixture (100 mL, 30%, v/v) was poured into the upper plastic tube. Water quickly passed through the filter and dropped into the beaker below， whereas oil was intercepted and kept in the upper tube. Pure water was successfully separated from the mixture, and no oil was observed in the collected water. After an oil-water separation cycle had finished, restarting the oil-water separation process by adding new water to the upper tube. The newly introduced water passed through the filter without any oil permeating through the filter. The separation process can therefore be repeated a number of times, and the separation filter can be used continuously. This experiment indicated that our filter is capable of separating a large amount of oil-water mixtures.
